# Personalized Treatment Modalities for Rectal Cancer: Advances in Neoadjuvant Treatment

**DOI:** 10.3390/jcm14134411

**Published:** 2025-06-20

**Authors:** Nussara Pakvisal, Leontios Pappas, Bennett A. Caughey, Rocco Ricciardi, Aparna Parikh

**Affiliations:** 1 Division of Hematology/Oncology, Mass General Brigham Cancer Center, Harvard Medical School, Boston, MA 02114, USA; npakvisal@mgh.harvard.edu (N.P.); lpappas3@mgb.org (L.P.); bcaughey1@mgb.org (B.A.C.); 2 Division of Medical Oncology, Department of Medicine, Faculty of Medicine, Chulalongkorn University and The King Chulalongkorn Memorial Hospital, Bangkok 10330, Thailand; 3 Section of Colon & Rectal Surgery, Massachusetts General Hospital, Harvard Medical School, Boston, MA 02114, USA; rricciardi1@mgh.harvard.edu

**Keywords:** locally advanced rectal cancer, neoadjuvant treatment, personalized treatment, total neoadjuvant therapy

## Abstract

Locally advanced rectal cancer treatment has shifted toward personalized, risk-adapted strategies that balance oncologic control with functional preservation while minimizing toxicity. A multidisciplinary team approach is essential, tailoring treatment guided by individual patient risk factors and priorities. Traditional neoadjuvant chemoradiation and subsequent total mesorectal excision has improved local control, but concerns remain regarding systemic failure and treatment-related morbidity. Total neoadjuvant therapy is now widely considered a preferred approach for more advanced tumors, enhancing systemic control, improving chemotherapy compliance, and facilitating organ preservation in select patients. Recent studies highlight that response-based treatment adaptation allows for better patient stratification, with selected patients who respond well to preoperative chemotherapy potentially omitting radiation without compromising outcomes and omitting surgery for patients with complete clinical responses to chemoradiation and chemotherapy. Advances in molecular profiling, particularly in mismatch repair deficiency or microsatellite instability-high tumors, have enabled the implementation of immune checkpoint inhibitors, permitting select patients to avoid both radiation and surgery, thereby reducing treatment-related toxicities. Future research should focus on validating predictive biomarkers, such as circulating tumor DNA, refining patient selection, and optimizing treatment monitoring while also developing novel therapeutic strategies to further personalize locally advanced rectal cancer management.

## 1. Introduction

Colorectal cancer has the fourth highest incidence among cancers in the United States (US) population with approximately 154,270 new patient cases and the second highest cause of mortality due to cancer in the US, with an estimated 52,900 deaths in 2025 underscoring its significant burden [1]. Rectal cancer comprises about one-third of these patients, with the majority presenting as locally advanced disease [2]. Notably, there has been a concerning rise in early-onset rectal cancer, with incidence rates increasing among individuals younger than 50 years old [3]. Recent epidemiological studies indicate that colorectal cancer is now a key driver of cancer-related mortality in young men [4]. In this younger population, the potential for long-term morbidity is particularly significant given their extended life expectancy and the impact of treatment-related side effects on quality of life [5].

The approach to the management of locally advanced rectal cancer (LARC) has progressed dramatically. Historically, surgery with proctectomy was the primary treatment, with a locoregional recurrence rate (LRR) of approximately 10% [6]. To further improve local control, total mesorectal excision, the addition of preoperative radiation, (both short-course radiation therapy (SCRT), and long-course chemoradiation (LCCRT)) were incorporated into treatment strategies [6,7,8,9]. However, recurrent distant metastases remained a concern, leading to the inclusion of systemic chemotherapy in the treatment sequence. Previously, chemotherapy was administered as postoperative/adjuvant therapy, but adherence rates were low and overall survival (OS) did not significantly improve [10]. Total neoadjuvant therapy (TNT), which incorporates neoadjuvant chemotherapy alongside either SCRT or LCCRT, has recently become the preferred approach in LARC to increase compliance with chemotherapy, enhance tumor regression, improve systemic disease control, and potentially lead to better long-term outcomes and organ preservation [11,12,13,14,15,16].

Disease management with multiple modalities may ultimately result in significant treatment-related toxicities and potentially long-term functional consequences, including the long-term impact on bowel, bladder, and sexual function [17,18]. As a result, more focused personalized treatment has become increasingly popular as an approach, balancing oncologic treatment with quality of life. This review explores advances in the treatment of LARC and the shift toward more tailored, risk-adapted management paradigms, with a focus on pivotal trials and key studies selected for their clinical relevance and impact on current practice.

## 2. Key Considerations in Personalized Treatment Modalities in LARC

### 2.1. Tumor Characteristics

Tumor characteristics play a fundamental role in guiding treatment decisions in LARC. Upon diagnosis, the initial workup typically includes computed tomography (CT) scans of the chest, whole abdomen, and pelvis to evaluate disease extent and rule out distant metastasis [19]. However, CT alone is insufficient for comprehensive local rectal staging [20,21]. Pelvic magnetic resonance imaging (MRI) is the preferred imaging modality for accurate tumor characterization, particularly for determining clinical T and N staging, an evaluation of the mesorectal fascia (MRF), and an evaluation for extramural vascular invasion (EMVI) [20,22,23,24,25]. In patients for whom MRI is contraindicated, endorectal ultrasound (ERUS) may serve as an alternative, particularly for assessing early T and N stage disease [26].

#### 2.1.1. Tumor Staging (Clinical T and N Stage)

Tumor staging determines the extent of the primary tumor and locoregional lymph node (LN) involvement, both of which influence treatment intensity. Patients classified as LARC have an elevated risk of local recurrence, necessitating neoadjuvant treatment [27]. There is no universal international alignment on the definition of LARC, but it is typically classified as clinical stages II or III [27], in which tumors extend across the muscularis propria into pericolorectal tissues (T3) or across the visceral peritoneum, potentially invading adjacent organs or structures (T4) and/or involving locoregional LN without distant metastasis [28].

A higher T stage, particularly T4b tumors with adjacent organ invasion, carries a higher risk of local recurrence [29], while a higher N stage, particularly N2 disease (≥4 positive locoregional LN) and lateral LN involvement, significantly elevate the risk of distant metastasis [30,31]. These staging factors play a critical role in treatment sequencing, helping clinicians determine whether systemic chemotherapy or local treatment, such as chemoradiation, should be the initial approach to balance local and distant disease control.

#### 2.1.2. Tumor Location

Tumor location is also an important consideration in treatment decision-making, as it influences both the likelihood of local recurrence and the extent of surgical intervention required [32]. Usually, a cancer is classified as rectal when the tumor is stationed up to 15 cm from the anal verge [33], but its specific location within the pelvis significantly impacts treatment planning. Some cases of higher rectal tumors located above the peritoneal reflection with peritoneal coverage have a lower risk of locoregional recurrence, acting more like colon cancer. As a result, perioperative radiation may be less important in these cases, particularly with good response to chemotherapy for less advanced cancers [34,35,36].

While mid- and lower-rectal tumors lack peritoneal coverage and are confined within the mesorectum, they are more challenging to resect completely, leading to a greater risk of local recurrence following surgery [37,38]. Therefore, neoadjuvant treatment is essential to mitigate this risk. For low-lying tumors near or involving the sphincter complex, preoperative SCRT alone is often insufficient for the tumor shrinkage needed for sphincter-preserving surgery and can result in a high risk of local failure [39]. The PROSPECT trial, which evaluated the omission of preoperative chemoradiation (CRT) in good responders to chemotherapy, excluded patients whose disease was not deemed appropriate for sphincter-sparing surgery, likely due to their higher risk of local recurrence without radiation [36]. Given these factors, most LARC patients with low-lying tumors require preoperative LCCRT to improve local control.

Additionally, tumors extending close to or involving the MRF pose a greater risk of an involved circumferential resection margin (CRM), a well-established predictor of local recurrence [40,41,42]. These patients frequently require preoperative LCCRT to facilitate downstaging and improve resectability.

#### 2.1.3. Extramural Vascular Invasion

EMVI refers to the localization of cancer cells within blood vessels, detectable on MRI, and it correlates with a greater risk of hematogenous spread and distant metastases [43]. Unlike other local tumor factors, EMVI primarily contributes to systemic rather than local recurrence [44]. Therefore, EMVI-positive LARC patients may benefit from preoperative systemic chemotherapy to reduce metastatic risk before definitive local treatment.

### 2.2. Molecular Characteristics

The emergence of molecular profiling has significantly influenced treatment decisions in rectal cancer. The most impactful molecular marker is mismatch repair deficiency (dMMR) or microsatellite instability-high status (MSI-H), which is found in 5–10% of all rectal adenocarcinomas [45] but only approximately 1% of LARC cases [46]. These tumors have shown poor responses to neoadjuvant chemotherapy in LARC, prompting the exploration of immune checkpoint inhibitors as an alternative approach [47].

A landmark study by Cercek et al. evaluated neoadjuvant dostarlimab, a programmed death-1 (PD-1) inhibitor, in dMMR/MSI-H LARC patients [45]. Among 41 patients who received 6 months of dostarlimab treatment, all were found to have a clinical complete response (cCR), with no need for further therapy or evidence of recurrence. At a median follow-up of 28.9 months, 20 patients maintained their complete response [48]. Given these findings, MSI/MMR testing is increasingly considered mandatory in LARC management, enabling personalized treatment by selecting immunotherapy candidates and supporting de-escalation strategies. A large multicenter study reported a 1.6% rate of discrepancy between immunohistochemistry (IHC) for dMMR and PCR-based MSI testing in colorectal cancer, primarily due to IHC misclassification [49]. Another study found that ~6% of MSI-H cases still expressed mismatch repair proteins on IHC [50], highlighting the potential for false negatives when relying solely on IHC. While dual testing is ideal, it may not always be feasible. In such cases, MSI testing is preferred when available. However, access to testing can be non-uniform, particularly in resource-limited settings, delaying patient ascertainment and potentially impacting care. Challenges such as inadequate insurance coverage or reimbursement and limitations in molecular testing facilities can further hinder such practices. Expanding access, establishing standard procedures, and incorporating cost-effective strategies are important for integrating MSI/MMR testing into routine practice.

### 2.3. Patient-Related Factors

Personalized treatment in LARC requires attention to individual patient characteristics independent of tumor biology that can significantly influence therapy selection. Symptoms, age, comorbidities, functional status, continence, and patient preferences play an important role in balancing quality of life with control of the underlying malignancy. The rising incidence of rectal cancer in young people [3,4] further underscores the need to preserve long-term organ function alongside survival outcomes.

#### 2.3.1. Patient Symptoms

A patient’s symptoms at presentation can impact both treatment sequencing and tolerability. Obstructive symptoms may necessitate early intervention with a diverting stoma before proceeding with systemic therapy or radiation [51]. In patients with significant local symptoms, such as intractable pain or persistent rectal bleeding, local treatment, including radiation, may be preferred as an upfront approach. Thus, symptom burden must be carefully evaluated when planning treatment sequencing to ensure optimal oncologic and functional outcomes.

#### 2.3.2. Age, Comorbidities, and Functional Status

Age, comorbidities, and baseline functional status determine a patient’s ability to tolerate aggressive treatments, particularly TNT strategies. In the PRODIGE 23 study, which evaluated induction chemotherapy before standard preoperative LCCRT and surgery, there were serious adverse events in up to 48% of patients, including 17% that experienced severe neutropenia, with granulocyte colony-stimulating factors required in 27% during induction with modified FOLFIRINOX, despite all participants having a WHO performance status of 0 or 1 [12]. These findings highlight the importance of careful patient selection to minimize toxicity while at the same time maximizing patient clinical outcomes.

Patients who are older or more frail face higher risks of morbidity and toxicity from chemotherapy, radiation, and surgery, which may require modification in treatment regimens and therapy de-escalation approaches. Conversely, younger patients require special attention to treatment-related functional outcomes given the significant implications of long-term alterations in bowel, urinary, and sexual function on quality of life. In light of their longer life expectancy, reducing long-term treatment toxicity and preserving function is a key priority. Additionally, younger survivors are at a higher risk of developing secondary cancers due to the long-term effects of radiation and chemotherapy [52,53,54]. As a result, de-escalation strategies, such as omitting preoperative radiation or organ preservation approaches in well-selected patients, have evolved to minimize unnecessary morbidity while maintaining good oncologic results [16,36].

#### 2.3.3. Patient Preferences

Treatment decisions in the modern era should increasingly align with patient priorities, balancing cancer control and functional organ preservation. While some patients and providers may prioritize survival with aggressive therapy, others may focus on quality of life and avoiding permanent stomas. Shared decision-making is essential for personalized care, including discussions on the aims of treatment, treatment side effects, functional outcomes, and impacts on lifestyle [55].

### 2.4. Risk-Adapted Treatment Strategies

**Figure 1:**
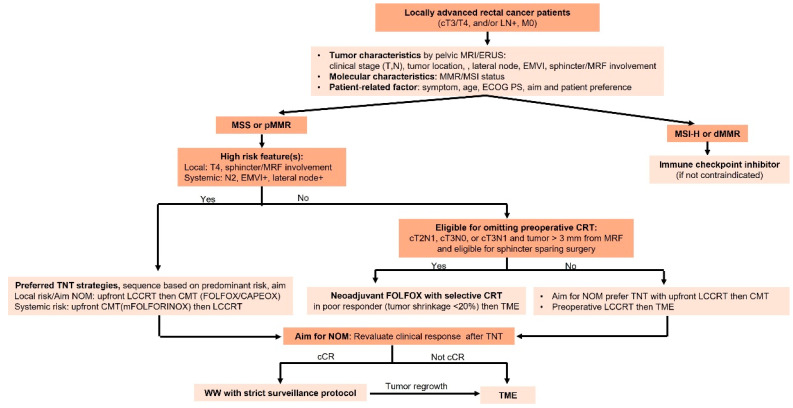
Treatment algorithm for personalized management in LARC. ERUS: endoscopic rectal ultrasound; EMVI: extramural vascular invasion; MRF: mesorectal fascia; MMR: mismatch repair; MSI: microsatellite instability; ECOG PS: Eastern Cooperative Oncology Group Performance status; CRT: chemoradiation; TNT: total neoadjuvant therapy; NOM: non-operative management; LCCRT: long-course chemoradiation; CMT: chemotherapy; cCR: clinical complete response; WW: watch and wait; TME: total mesorectal excision.

Consideration of the factors discussed above can lead to risk-adapted treatment strategies in LARC that may allow for treatment intensification in high-risk patients to improve oncologic control, as well as treatment de-escalation in good responders to minimize long-term toxicity and preserve function, as shown in Figure 1. Restaging after neoadjuvant therapy is crucial for determining the appropriate next steps, particularly in the context of non-operative management. A multimodality assessment—including clinical evaluation, digital rectal examination, endoscopic findings, CT scans of the chest and abdomen, and high-resolution pelvic MRI with diffusion-weighted imaging—enhances the accuracy of distinguishing complete, near-complete, and incomplete responses. Accurate response classification safely guides treatment decisions and supports a personalized approach.

#### 2.4.1. Treatment Intensification

TNT integrates systemic chemotherapy and radiation/CRT prior to surgery. It is a preferred approach for high-risk LARC as it improves systemic disease control, enhances tumor downstaging, and increases resectability [56]. TNT is particularly beneficial for patients with N2 disease or EMVI positivity to improve systemic control, while those with T4 disease or MRF involvement benefit from better tumor regression [11,12,13].

The RAPIDO trial was among the first randomized phase III studies evaluating the TNT approach. It compared neoadjuvant SCRT and subsequent systemic chemotherapy (nine cycles of FOLFOX or six cycles of CAPOX) before surgery with the conventional approach of preoperative LCCRT, followed by adjuvant chemotherapy. Patients enrolled in the study had at least one high-risk feature on pelvic MRI, including cT4 stage, cN2, EMVI, positive MRF, or enlarged lateral lymph nodes. The study demonstrated that the experimental TNT approach significantly reduced the frequency of disease-related treatment failure at three years (23.7% vs. 30.4%), primarily due to fewer distant metastases. Additionally, pathological complete responses (pCR) were higher in the experimental TNT cohort (28.4% vs. 14.3%, *p* < 0.001), indicating improved tumor regression prior to surgery [13]. However, with an extended follow-up of 5.6 years, the LRR was found to be higher in the TNT cohort than in the preoperative LCCRT cohort (10% vs. 6%, *p* = 0.027). Further analysis identified key risk factors for locoregional recurrence to include enlarged lateral LN, positive CRM, tumor deposits, and LN involvement [57]. Though RAPIDO was pivotal in popularizing the use of TNT in routine practice, these findings emphasize the need for careful patient selection and close monitoring when utilizing this treatment strategy in the clinic.

The PRODIGE 23 study, a phase III randomized trial, compared TNT with six cycles of modified FOLFIRINOX as induction chemotherapy before standard LCCRT and surgery, followed by adjuvant FOLFOX or capecitabine, to neoadjuvant LCCRT and adjuvant chemotherapy for 6 months in patients diagnosed with cT3 or cT4 LARC. The results showed a significant increase in three-year disease-free survival (DFS) (75.7% vs. 68.5%, HR = 0.69, *p* = 0.034) with the TNT approach, along with a greater pCR rate (28% vs. 12%, *p* < 0.001) and better metastasis-free survival (MFS) (78.8% vs. 71.7%, HR = 0.64, *p* = 0.017). Importantly, no statistically significant difference in LRR was observed (4% vs. 6%, HR = 0.78, *p* = 0.56) [12]. Long-term follow-up (median 82.2 months) confirmed DFS and MFS benefits without increasing the risk of locoregional failure and showed a significant improvement in seven-year OS (81.9% vs. 76.1%, *p* = 0.033) [58]. These findings support TNT with induction-modified FOLFIRINOX as an effective approach for patients with high-risk LARC, delivering systemic chemotherapy earlier to improve systemic control without compromising local control.

Beyond its role in high-risk LARC patients, TNT has also been used as a platform for treatment de-escalation, particularly in organ preservation strategies, which are discussed below.

#### 2.4.2. Treatment De-Escalation

In contrast to intensification strategies, the goal of treatment de-escalation is to decrease treatment-related toxicities while preserving oncologic outcomes. This approach is especially important for patients with favorable tumor characteristics or those achieving an excellent response to neoadjuvant therapy.

#### 2.4.3. Omitting Preoperative Radiation

Although preoperative CRT has been a key component of standard-of-care treatment for LARC, recent trials have investigated preoperative systemic therapy alone to minimize radiation-related toxicity affecting quality of life [59] while maintaining anti-cancer benefits [36,45]. This approach is particularly relevant for patients with a lower biological risk profile and good response to systemic treatment, including chemotherapy or immunotherapy [36,45].

The PROSPECT trial, a randomized noninferiority study, evaluated whether preoperative FOLFOX could replace preoperative CRT in patients with clinical stage T2N1, T3N0, or T3N1 rectal cancer and candidates for a sphincter-sparing operation. Tumor staging was determined through physical examination, CT imaging, and either pelvic MRI or endorectal ultrasound, with exclusion criteria including tumors located within 3 mm of the MRF. Patients’ randomization was 1:1 to either conventional preoperative CRT or six cycles of modified FOLFOX6, with CRT selectively administered if tumors exhibited less than a 20% reduction in size prior to surgery. Postoperative adjuvant chemotherapy, consisting of an additional six cycles of FOLFOX, was advised but not mandatory. Ultimately, 75% of patients in the FOLFOX cohort and 78% in the CRT group received adjuvant chemotherapy. Following a 58-month median follow-up, the study found that preoperative FOLFOX with selective CRT was noninferior to standard CRT with respect to DFS (HR = 0.92, 90.2% CI: 0.74–1.14, *p* = 0.005 for noninferiority), demonstrating 5-year DFS rates of 80.8% and 78.6%, respectively. Pathologic outcomes were also comparable between the groups, with pCR rates of 21.9% in the preoperative chemotherapy group and 24.3% in the preoperative CRT group. There were no R2 resections and only 1.1% of cases had R1 resection among patients who received preoperative FOLFOX. Moreover, there were no statistically significant differences in 5-year LRR (1.8% vs. 1.6%) and OS (89.5% vs. 90.2%) between the two cohorts [36].

Notably, 89.6% of patients in the preoperative FOLFOX group avoided radiation, reducing the risk of radiation toxicity [36]. However, careful patient selection is crucial. The pretreatment evaluation and clinical staging in PROSPECT relied on high-sensitivity imaging (pelvic MRI or ERUS) and multidisciplinary decision-making, including surgeon assessment to determine eligibility for sphincter-sparing surgery. Additionally, tumor response assessment (≥20% reduction) was determined using restaging imaging, proctoscopy, and physical examination [36], emphasizing the need for high-quality imaging and a well-coordinated multidisciplinary team approach.

Regarding neoadjuvant immune checkpoint inhibitors without preoperative CRT, studies have shown promising tumor control in selected dMMR/MSI-H patients [48], as discussed in the molecular characteristics section. These findings suggest that systemic treatment alone, with the selective use of CRT, enables treatment de-escalation based on tumor response, offering a more personalized approach to LARC management. Moving forward, identifying optimal selection criteria for preoperative chemotherapy and refining response-based treatment adaptations will be crucial to maintaining oncologic outcomes while minimizing treatment-associated morbidity.

#### 2.4.4. Non-Operative Management (NOM)/Watch and Wait (WW)

While TME remains the standard surgical approach, it can result in significant morbidity, including bowel, urinary, and sexual dysfunction [17]. Additionally, low-lying tumors often necessitate a permanent colostomy, affecting quality of life [17]. One of the key objectives of TNT in LARC treatment has been to enable organ preservation in selected patients without compromising oncologic outcomes [16].

The OPRA trial, a randomized phase II study, evaluated stage II–III rectal cancer patients treated with TNT using either CRT and subsequent consolidation chemotherapy (CRT-CNCT) or induction chemotherapy and subsequent CRT (INCT-CRT). The Watch and Wait (WW) approach was pursued in patients who had either a clinical complete response (cCR) or near-clinical complete response (ncCR). The primary endpoint—whether TNT incorporating an outcome-driven WW approach improved DFS compared to historical controls—was not met, as both groups had a three-year DFS of 76% [16]. However, with a 5.1 years median follow-up, the CRT-CNCT group showed a superior five-year organ preservation rate compared to the INCT-CRT group (TME-free survival rate: 54% vs. 39%, *p* = 0.012) [60], likely due to a greater time span between CRT completion and tumor restaging, increasing the likelihood of tumor regression. Despite these benefits, 36% of patients under WW experienced tumor regrowth. Among patients with local regrowth, 94% were detected within two years and 99% by the end of three years, reinforcing the need for intensive surveillance [60]. The OPRA surveillance schedule consisted of

- Flexible sigmoidoscopy and digital rectal examination (DRE) conducted every four months during the initial two years, followed by assessments every six months for the subsequent three years.
- Pelvic MRI performed every six months for the initial two years, transitioning to annual imaging thereafter.

This structured follow-up protocol facilitates early tumor regrowth detection. When local regrowth is detected, salvage surgery is the preferred approach. The OPRA trial found that patients who had salvage TME after tumor regrowth demonstrated comparable DFS to those who were treated with upfront TME, with 5.1 years median follow-up time [60]. These findings support that WW does not result in inferior long-term outcomes in patients who undergo intensive surveillance schedules and timely salvage surgery upon recurrence. Regardless, a careful analysis of longer-term outcomes will be essential to fully assess the effectiveness of the WW strategy as opposed to upfront planned surgery.

As organ preservation strategies evolve, challenges remain in identifying optimal candidates. Advances in imaging techniques and biomarker-driven selection may improve patient selection for nonoperative management. The decision to pursue WW should be made within a multidisciplinary team setting, considering individual patient preferences, tumor biology, and the feasibility of long-term follow-up. Moving forward, refining patient selection criteria and optimizing surveillance strategies will be essential to ensuring that organ preservation approaches maintain good anti-cancer outcomes while improving quality of life in LARC patients (Table 1).

### 2.5. Multidisciplinary Team Involvement

The optimal management of LARC necessitates a multidisciplinary team (MDT), comprising radiologists, pathologists, surgeons, and medical and radiation oncologists to tailor treatment based on individualized patient factors. MDT discussions play a crucial role in accurately staging T and N classifications, assessing high-risk MRI features—such as EMVI and MRF involvement—and formulating treatment plans [61,62]. Collaboration among specialists ensures a personalized approach, integrating all key considerations discussed above to optimize oncologic control and functional outcomes.

## 3. Emerging Trends and Future Directions

### 3.1. Intensified Chemotherapy to Avoid Radiation and Enhance NOM in LARC

TNT has evolved into standard-of-care practice for LARC, particularly in patients with high-risk features. The PRODIGE 23 trial demonstrated that starting treatment with modified FOLFIRINOX, a high-intensity chemotherapy regimen, can lead to high response rates [12]. These findings raise critical clinical questions:

(1) Can good responders after preoperative FOLFIRINOX safely omit preoperative CRT, such as in the PROSPECT trial explored in patients receiving FOLFOX?

The GRECCAR 14 study, an ongoing phase II-III non-inferiority trial, is designed to explore this approach in higher-risk LARC patients. It includes patients with mid- or lower-rectal tumors with a CRM ≤ 2 mm (predicted) or T3c-d stage with EMVI. Tumor response is evaluated after six cycles of FOLFIRINOX, and good responders (≥60% tumor volume reduction on MRI) are then treated with either preoperative CRT or surgery, with 1:1 randomization. Patients with pathological staging higher than ypT2 or ypN1 are recommended to pursue adjuvant chemotherapy. The study’s primary endpoints are the frequency of an R0 resection for the phase II portion and 3-year DFS for the phase III portion [63]. GRECCAR 14 will provide valuable insights into omitting preoperative CRT in high-risk LARC patients after intensified chemotherapy, addressing a population with more aggressive tumor features than those studied in PROSPECT. If successful, this trial could further personalize treatment strategies, reducing unnecessary toxicity while maintaining oncologic outcomes.

(2) Will a consolidation triplet regimen with FOLFORINOX achieve higher cCR rates and improve organ preservation than a doublet regimen followed by CRT in a TNT strategy?

The Alliance A022104 trial, an ongoing randomized phase II/III study, addresses this question. Patients with LARC (including T4N0, any T and N positive, and T3N0 needing abdominoperineal resection or a coloanal anastomosis) are randomly assigned to undergo neoadjuvant LCCRT followed by consolidation chemotherapy with either a doublet regimen (mFOLFOX6/CAPOX) or a triplet regimen (mFOLFORINOX) for 3–4 months. The study’s primary endpoints include cCR for phase II and DFS for phase III [64]. If the trial validates its hypotheses, intensified chemotherapy could improve cCR and DFS rates, increase the possibility of organ preservation, and enhance quality outcomes for LARC patients.

### 3.2. Role of Circulating Tumor DNA in Personalizing Non-Operative Strategies

NOM is a feasible approach for selected patients with LARC; however, patient selection and disease monitoring remain significant challenges. Criteria for selecting patients for WW strategies vary across clinical trials, but a common requirement is achieving a cCR based on DRE, MRI, and endoscopic findings after completing TNT [65,66]. However, a cCR does not necessarily correspond to a pCR [65], and up to 22% of patients with initial cCR in the OPRA trial experienced tumor regrowth [60].

In this context, identifying biomarkers to predict pCR in patients with cCR could refine the selection of patients for a WW approach [67]. One such candidate biomarker is circulating tumor DNA (ctDNA), which is evolving into a promising instrument for predicting outcomes in early-stage colon cancer [68]. Several studies have investigated and found that ctDNA clearance after neoadjuvant CRT (nCRT) in LARC can be an independent predictor for pCR, particularly when integrated with MRI findings [69,70,71]. Additionally, detectable presurgery ctDNA after TNT has been significantly associated with systemic recurrence, shorter DFS, and OS [72]. Recent preliminary data from a small cohort of 28 cases suggest that post-TNT ctDNA status may serve as a predictor of both clinical and pathological response in LARC [73]. Despite its potential, several limitations exist in current clinical practice. First, most reported studies have investigated ctDNA in the setting of nCRT; there is limited data on TNT. Second, no studies have reported the prediction of tumor regrowth or frequency of local recurrence in patients with positive ctDNA after TNT within the WW context. Lastly, there is no standardization of ctDNA testing with variations in assay sensitivity, interpretation, and clinical validation [74]. Thus, larger multicenter studies would be required to validate the role of ctDNA in guiding non-operative management and treatment personalization. Future research should focus on integrating ctDNA with conventional imaging and other molecular markers to enhance predictive accuracy, improve patient stratification, and ultimately optimize risk-adapted treatment approaches for LARC (Table 2).

## 4. Conclusions

The treatment of LARC is becoming increasingly personalized, aiming to balance oncologic control with functional preservation. Our proposed algorithm (Figure 1) integrates key considerations to guide neoadjuvant strategies based on individual risk. Advances in response-based adaptation, organ preservation, and emerging biomarkers such as ctDNA are driving more precise, risk-adapted management, improving both survival and quality of life.

## Figures and Tables

**Table 1:** Summary of key clinical trials in neoadjuvant treatment for locally advanced rectal cancer.

Trial Name	Study Design	Population	Treatment Arm	Primary Endpoint	Key Findings
Cercek et al. [45]	Prospective phase 2	dMMR/MSI-H stage II or III	Neoadjuvant Dostarlimab × 6 months then restaging if cCR → Watch and Wait if non cCR → LCCRT then TME	cCR at 12 months after Dostarlimab	100% cCR
RAPIDO [13]	RCT phase 3	≥1 high-risk feature: cT4 or cN2, EMVI, positive MRF, or lateral LN	SCRT → FOLFOX × 9/CAPEOX × 6 → TME vs. LCCRT → TME → with/without FOLFOX × 12/CAPEOX × 8	3-year DRTF	23.7% vs. 30.4%
PRODIGE -23 [12]	RCT phase 3	cT3 or cT4	FOLFORINOX × 6 → LCCRT → TME → FOLFOX × 6/Capecitabine vs. LCCRT → TME → FOLFOX × 6/Capecitabine	3-year DFS	75.7% vs. 68.5%
PROSPECT [36]	RCT, noninferiority study	cT2N1, cT3N0, or cT3N1 and candidates for sphincter-sparing surgery	Preoperative FOLFOX × 6 → Restaging if ≥20% response → TME then adjuvant CMT if <20% response → LCCRT → TME then adjuvant CMT vs. LCCRT → TME then adjuvant CMT	DFS	HR 0.92 (90.2% CI: 0.74–1.14) *p* = 0.005
OPRA [16]	RCT phase 2	stage II or III	FOLOX × 8/CAPEOX × 5 → LCCRT vs. LCCRT → FOLOX × 8/CAPEOX × 5 restaging both arms if cCR/ncCR → Watch and Wait if incomplete response → TME	DFS	3-year DFS 76% in both groups

dMMR: deficient mismatch repair; MSI-H: microsatellite instability-high; cCR: clinical complete response; RCT: randomized controlled trial; c: clinical; EMVI: extramural vascular invasion; MRF: mesorectal fascia; LN: lymph node; SCRT: short-course radiation therapy; TME: total mesorectal excision; LCCRT: long-course chemoradiation; DRTF: disease-related treatment failure (first locoregional recurrence, distant metastasis, new primary tumor, or death because of treatment); DFS: disease-free survival; CMT: chemotherapy; HR: hazard ratio; CI: confidence interval; CR: complete response; ncCR: near-clinical complete response; vs.: versus.

**Table 2:** Summary of ongoing clinical trials investigating ctDNA in locally advanced rectal cancer.

Trial ID	Study Design	Population	Treatment/Intervention	Primary Endpoint	ctDNA Application
NCT05601505 (CINTS-R) [75]	RCT phase 2	stage II or III	-Control: nCRT then TME -Experimental: if baseline ctDNA VAF < 0.5% → nCRT, VAF ≥ 0.5% or +post-CRT ctDNA → TNT then TME	2-year DRTF	ctDNA-guided neoadjuvant treatment strategies
NCT04842006 (SYNCOPE) [76]	RCT	stage II or III with EMVI	-Control: LCCRT then TME -Experimental: TNT with SCRT then CAPEOX then TME	RFS	Postoperative ctDNA for MRD assessment and its correlation with RFS
NCT03714490 (SUNRISE) [77]	RCT phase 2	stage II or III with cT4b or positive MRF	-Control: LCCRT then TME -Experimental: TNT with SCRT then CAPEOX then TME	R0 resection rate	Correlation of ctDNA clearance or persistence with treatment response and DFS/OS
NCT05081024 (ctTRAC) [78]	Prospective observational cohort study	stage II or III planned for TNT	Blood collection for ctDNA at baseline, every 2 months while undergoing TNT, and then every 3 months for up to 3 years after completion of TNT	cCR rate	ctDNA monitoring as biomarker to predict cCR after TNT
NCT05674422 (REVEAL) [79]	Prospective observational cohort study	stage II or III planned for TNT then WW if cCR or ncCR	Blood collection for ctDNA up to 2 years after starting TNT	PPV and NPV of post-TNT ctDNA to identify relapses in 2 years after TNT	ctDNA monitoring as biomarker to predict relapse after TNT
NCT06364371 [80]	Prospective observational cohort study	stage II or III planned for neoadjuvant treatment	Monitoring MRI scans, histopathology slides, CEA and ctDNA at pre-, during, and post-treatment	Area under curve of prediction model for predicting pCR	ctDNA monitoring as biomarker to predict pCR

ctDNA: circulating tumor DNA; RCT: randomized controlled trial; nCRT: neoadjuvant chemoradiation; TNT: total neoadjuvant therapy; TME: total mesorectal excision; VAF: variant allele fraction EMVI: extramural vascular invasion; LCCRT: long-course chemoradiation; SCRT: short-course radiation therapy; MRD: minimal residual disease; RFS: recurrence-free survival; DFS: disease-free survival; OS: overall survival; cCR: clinical complete response; ncCR: near-clinical complete response; PPV: positive predictive value; NPV: negative predictive value; MRI: magnetic resonance imaging; CEA: carcinoembryonic antigen; pCR: pathological complete response; WW: Watch and Wait.

## Data Availability

Not applicable.
